# Complete Genome Sequence of a Fish Nervous Necrosis Virus Isolated from Sea Perch (*Lateolabrax japonicus*) in China

**DOI:** 10.1128/genomeA.00048-15

**Published:** 2015-06-04

**Authors:** Peng Jia, Kun-Tong Jia, Mei-Sheng Yi

**Affiliations:** School of Marine Sciences, Sun Yat-sen University, Guangzhou, Guangdong, China

## Abstract

We sequenced and analyzed the complete genome of a fish nervous necrosis virus isolated from diseased sea perch (*Lateolabrax japonicus*) in Guangdong Province, China. The virus genome contains RNA1 (3,103 bp) and RNA2 (1,433 bp). Phylogenetic analysis shows that the virus belongs to the redspotted grouper nervous necrosis virus genotype of betanodavirus.

## GENOME ANNOUNCEMENT

Viral nervous necrosis (VNN) has already become a serious problem in several farmed marine and freshwater fish species in various geographic areas all over the world (1). Currently, more than 50 fish species, including members of the *Anguilliformes*, *Gadiformes*, *Perciformes*, *Pleuroneetiformes*, and *Tetraodontiformes*, have been reported to be affected by VNN (2). The disease can transmit both horizontally via water and vertically via reproduction. It usually occurs in larvae or juveniles resulting in high mortality rates of more than 90%, often up to 100% (3). Nervous necrosis virus (NNV) of fish, also called betanodavirus, is the causative agents of VNN which consists of two coencapsidated positive-sense RNA segments without poly(A) structure at the 3′ terminus. RNA1 encodes for the RNA-dependent RNA polymerase (RdRp) which replicates the viral genomes, while RNA2 encodes for a precursor to the coat protein (CP) (4). A subgenomic RNA3 encoding for nonstructural viral proteins B1 and B2 is synthesized during virus replication (5, 6). Based on the CP sequence, betanodaviruses are categorized into four different genotypes: TPNNV (tiger puffer nervous necrosis virus), SJNNV (striped jack nervous necrosis virus), BFNNV (barfin flounder nervous necrosis virus), and RGNNV (redspotted grouper nervous necrosis virus) (7).

Sea perch (*Lateolabrax japonicus*) larvae and juveniles naturally infected by NNV (strain SBN147) were collected from a fish farm in Guangdong Province, China. The sequences of RNA1 and RNA2 genes were PCR amplified by using two pairs of primers designed on the basis of known betanodavirus. Then, the PCR products were subcloned into PMD-19-T vector (TaKaRa, Japan) for sequencing.

The RNA1 is 3,103 nucleotides (nt) in length and contains an open reading frame (ORF) spanning from nt 79 to 3,027, encoding for a protein (RdRp) of 982 amino acid with a molecular mass of 110,396.68 Da, and with an isoelectric point (IP) of 8.278. The 5′ and 3′ untranslated regions (UTRs) are located from positions nt 1 to 78, and nt 3,028 to 3,103, respectively. The RNA2 sequence is 1,433 nt and contains an ORF between nt 27 and 1,043, encoding for a protein (CP) of 338 amino acid with a molecular mass of 37,060.88 Da, and with an IP of 8.867. The 5′ and 3′ UTRs are located from nt 1 to 26 and nt 1,044 to 1,433, respectively.

Phylogenetic tree analysis of the CP shows that strain SBN147 belongs to the RGNNV genotype of betanodavirus, and the CP gene from strain SBN147 had the highest identity of 99% with seven-band grouper nervous necrosis virus at nucleotide level. This genome information will provide useful information for understanding the relationship between betanodaviruses and sea perch (*Lateolabrax japonicus*) and betanodavirus pathogenesis.

### Nucleotide sequence accession numbers. 

The complete genome sequence of the NNV (strain SBN147) has been deposited in GenBank under the accession numbers KP455642 and KP455643.
